# Phytoplankton-Zooplankton Community Structure in Coal Mining Subsidence Lake

**DOI:** 10.3390/ijerph20010484

**Published:** 2022-12-28

**Authors:** Tingyu Fan, Hayat Amzil, Wangkai Fang, Liangji Xu, Akang Lu, Shun Wang, Xingming Wang, Yingxiang Chen, Jinhong Pan, Xiangping Wei

**Affiliations:** 1 School of Earth and Environment, Anhui University of Science and Technology, Huainan 232001, China; 2 Anhui Engineering Laboratory for Comprehensive Utilization of Water and Soil Resources & Ecological Protection in Mining Area with High Groundwater Level, Huainan 232001, China; 3 Huaibei Mining Group, Huaibei 235000, China

**Keywords:** subsidence lake, phytoplankton, zooplankton, community abundance, diversity, water parameters

## Abstract

Land subsidence from coal mining has shaped new artificial aquatic ecosystems, these subsidence lakes are known for their restricted ecological system, water pollution, and extreme habitat conditions. However, knowledge concerning the community structure of plankton in these types of water bodies is still limited. Therefore, both phytoplankton and zooplankton communities’ abundance, distribution, and diversity, as well as relations of these communities to physicochemical water quality variables were analyzed, alongside the interaction between phytoplankton and zooplankton groups. The results indicate zooplankton abundance was 842.375 to 186,355.0 ind./L. Biomass ranged from 0.3408 to 10.0842 mg/L. Phytoplankton abundance varied between 0.541 × 10^6^ cell/L and 52.340 × 10^6^ cell/L while phytoplankton wet biomass ranged from 0.5123 to 5.6532 mg/L. Pearson correlation analysis revealed that both the zooplankton and phytoplankton total densities were significantly correlated with nutrients (TN, TP, PO_4_^3−^) and COD_cr_; zooplankton abundance was significantly correlated with phytoplankton abundance. According to the biodiversity index of Shannon–Wiener, both phytoplankton and zooplankton revealed less biodiversity in the subsidence water region than in the Huihe river system and Xiangshun canal, with values ranging from 0.20 to 2.60 for phytoplankton and 1.18 to 2.45 for zooplankton; however, the phytoplankton community showed lower biodiversity index values compared to the zooplankton community. Overall, the knowledge gleaned from the study of plankton community structure and diversity represents a valuable approach for the evaluation of the ecological conditions within the subsidence lakes, which has significant repercussions for the management and protection of aquatic environments in mining areas.

## 1. Introduction

Coal is one of the most important energy sources in the world, and its exploitation and utilization are increasing year by year [1,2]. A large amount of coal mining has brought about a series of ecological and environmental problems [3]. Land subsidence due to coal mining operations is one of the critical factors affecting surface structures and water resources [4]. Excessive mining has led to ground displacement and deformation, and eventually the construction of a vast subsidence area, with low groundwater levels and rainfall. The original terrestrial environment then gradually evolves into an aquatic environment [5].

The quantities and qualities of these subsidence lakes were described, and recommendations for their ecological restoration were considered. Quantitatively, they are generally regulated as fishponds, wetland parks, and large plain reservoirs or ecological lakes based on the water bodies, scales, locations, and hydrology. However, the water qualities show different patterns and degrees of pollution [6]. As a result of their low topography, these water bodies may be polluted by mine water, leaching water from waste heaps, domestic sewage, and agricultural drainage [5,7].

The existence of coal mining activities, in addition to creating large ponds due to mining excavation, is also estimated to creature pressure on the surrounding aquatic ecosystem [8]. Certain subsidence water bodies affected by long-term rainfall and human activities continue to expand the scope of subsidence, and gradually connect with adjacent rivers, causing the enrichment of contamination sources in rivers [9].

In aquatic ecosystems, phytoplankton and zooplankton are important components, contributing to the ecosystem’s health and services [10]. Phytoplankton are the first bio-indicators of pollution in aquatic ecosystems [11]. Phytoplankton play a critical role in the structure and functioning of aquatic ecosystems. As primary producers, they are considered a significant component of water ecosystems [12]. Populations of phytoplankton are well known to be influenced by spatiotemporal variations in hydro-chemical and physical parameters [13,14], and they directly affect the water quality variables such as turbidity and dissolved oxygen, thus influencing many ecosystem processes [15]. The monitoring of phytoplankton and algae is of great significance because phytoplankton demonstrates the health of an aquatic ecosystem through changes in its community composition and distribution, and the proportion of sensitive species [16,17].

Zooplankton are a primary component of aquatic ecosystems that are included within the transformation of organic matter and the formation of matter and energy fluxes. The diversity and abundance of zooplankton species are sensitive indicators of anthropogenic changes in environmental conditions [18,19]. Zooplankton are characterized by short life cycles and fast adaptation to environmental changes [20]. They are sensitive to various substances in water such as nutrient enrichment and pollutants. Thus, they have often been used as indicators to assess the condition and change of the freshwater environment [21]. The relationships established by zooplankton with phytoplankton as the primary producers reflect the ecological conditions of the entire ecosystem [22].

Given the above information, the research of zooplankton and phytoplankton communities in relation to coal mining subsidence lake conditions were considered to be necessary. Abiotic and biotic factors of an ecosystem are interdependent, and the change of abiotic factors frequently affects the biotic factors, which will be reflected directly in their distribution, abundance, and diversity [23,24,25]. Therefore, it was assumed that the structure of zooplankton and phytoplankton communities is shaped under the influence of various abiotic and biotic factors [20,26,27]. Numerous related studies have been performed and stated that temperature, transparency (SD), and nutrient concentrations were important factors affecting the phytoplankton community [28,29], while nutrients, temperature, EC, TDS, and dissolved oxygen were major factors that restrict the abundance and diversity of the zooplankton communities [20,30]. However, these previous studies have mainly focused on natural freshwater ecosystems and there is still a lack of research on the community structure of phytoplankton and zooplankton in the coal mining subsidence lakes.

The Linhuan Mining Area is located in Suixi County, Huaibei City, Anhui Province of China; the Huihe river is a small to medium-sized seasonal river, which flows from west to east through this region. The Linhuan coal mining subsidence area is formed due to the coal mining subsidence. The accumulated water mainly comes from atmospheric precipitation and surface runoff, and the evaporation of surface water is the main excretion route. Part of the subsidence lake supplies water to the Linhuan Industrial Park, with the water surface covered with photovoltaic panels for solar power generation while the remaining part has been developed into a fish pond. The fly ash field and gangue hill are located to the east of the subsidence lake. To the southwest are the Linhuan Industrial Park and the small gangue hill. In the north, there is the Xiangshun canal (artificial diversion canal) connected to the Huihe River. The Huihe River is introduced into the subsidence lake as its water source [31]. With the progress of coal mining, the area of subsidence will be further increased. While this area’s ecological environment has shifted from a terrestrial to a water-land composite ecosystem, it is still impacted by acid wastewater from mines, agriculture, and domestic sewage. As a result, the ecosystem’s structure and function have been severely disrupted.

The present study, therefore, highlights the characteristics of both phytoplankton and zooplankton communities and their distribution, abundance, and diversity. It also assesses the trophic linkage between them and their relationships with the physicochemical parameters within the Linhuan subsidence lake in the Linhuan Mining Area, as well as the Huihe river area in Suixi County, Huaibei City, China. The outcome of this research will provide a better understanding of the Linhuan subsidence lake and the Huihe river as an important aspect of ecosystem management and environmental protection.

## 2. Materials and Methods

### 2.1. Study Area and Locations of Samples

The subsidence area is geographically located between 116°34′25″~116°44′27″ E and 33°36′50″~34°40′47″ N. The prevailing wind in the mining area is northeast in spring and autumn, mostly east to southeast in summer, and north to northwest in winter. The average wind speed, temperature and annual rainfall is 2.0 m/s, 14.1 °C, and 830.0 mm, respectively. The average water depth of the Linhuan Subsidence Lake is about 3.45 m, the maximum water depth is 9.0 m, and the accumulated water area is 5.5 km^2^. The water storage period exceeds 17 years [32].

**Figure 1:**
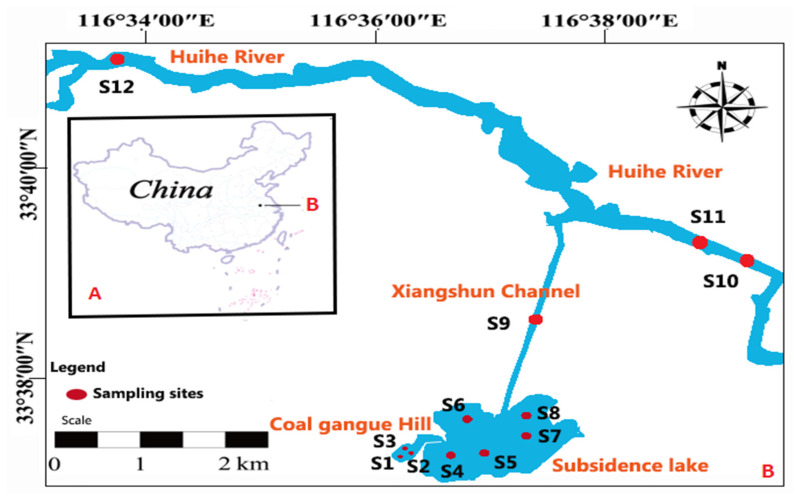
Study area and sampling points. (**A**) map of China, and (**B**) sampling sites in the coal mining subsidence lake and in the Huihe river.

In the present study, 12 sampling sites were set up in the coal mining subsidence in Linhuan Mining Area and Huihe River, along with hydrogeological conditions and on-site investigations. The coal mining subsidence lake (SL) includes five sampling points (S4, S5, S6, S7 and S8) and three sampling points (S1, S2, S3) in coal gangue hills (CGH). From upstream to downstream of the Huihe River (HR), three sampling points (S10, S11, and S12) were chosen, alongside one sampling point (S9) in Xiangshun canal. Figure 1 illustrates the distribution of the sampling points and Table S1 presents sampling point coordinates.

### 2.2. Samples Collection

The water samples were collected in April 2021 using pre-cleaned high-density polyethylene plastic bottles. For both the phytoplankton and zooplankton studies, Apstein plankton nets were used to collect samples along the water column by hauling vertically from the bottom to surface waters; due to the small depth, water samples for analysis were collected from 0.5 m below the water surface. The collected phytoplankton samples were preserved in 50 mL sterile polyethylene bottles and fixed with 10% of Ruger’s reagent, while the zooplankton samples were preserved in 50 mL polyethylene bottles and with 10% formalin. The samples were then transported to a bioinformatics company laboratory for identification and counting under a microscope.

### 2.3. Physicochemical Analysis

From each sampling point, basic parameters of water quality, such as water temperature (T), pH, total dissolved solids (TDS), electrical conductivity (EC), dissolved oxygen (DO), depth (H), and SD (Secchi depth) were determined in the field using a pH meter, Secchi disk, and multiparameter water quality sonde (YSI 6600 V2, Yellow Springs Instruments Inc., Yellow Spring, OH, USA).

Ions, Chemical Oxygen Demand (COD_cr_), and nutrients of the water samples were tested in a Bioinformatics company laboratory using analytical methods. The concentrations of anions (SO_4_^2−^, NO_3_^−^, F^−^, Cl^−^) were estimated using Ion Chromatography, while the concentrations of Chemical Oxygen Demand (COD_cr_) were determined using the Potassium Dichromate Method. For nutrients concentrations (TN, NH_4_^+^, TP, PO_4_^3−^), Total Nitrogen (TN) was determined using Alkaline potassium persulfate digestion UV spectrophotometry, ammonium ions (NH_4_^+^) were quantified using Nessler’s reagent spectrophotometry, and the concentrations of total phosphorus (TP) and orthophosphate (PO_4_^3−^) were measured by Ammonium molybdate spectrophotometric method.

### 2.4. Biological Data Preparation and Analysis

Identification and counting, species name, species number, individual number of each species, total number, biomass, and density estimation as well as diversity index estimation of phytoplankton and zooplankton at each sampling point were completed and recorded by a bioinformatics company named Shandong Xiaochong Biotechnology Co., Ltd. and located in Shandong, China. Taxonomic identifications were carried out at the species level.

The phytoplankton samples were concentrated from 1 L to 50 mL. Phytoplankton cells were enumerated using a counting cell of a volume of 0.1 mL and an area of 400 mm^2^, under a microscope, and the number of fields of view counted was 100 view fields; the area of each field of view was 0.158962 mm^2^ and the counting area was 15.8962 mm^2^.

The phytoplankton community cell density of each taxon was calculated as follows [33]:

*N* = *Cs* × *v* × *Pn*/(*Fs* × *Fn* × *V*) (1)

where *N* is the cell density, *Cs* is the area of the counting cell, *v* is the volume of the concentrated samples, *Pn* is the number of phytoplankton cells, *Fs* is the area of each view field, *Fn* is the number of view fields and *V* is the volume of the counting cell.

Zooplankton samples were concentrated from 1 L to 50 mL. Zooplankton were counted in 1 mL subsamples made with an automatic pipet after homogenization of the sample. Zooplankton densities were estimated based on the individuals found in the samples and the water samples’ volume, expressed in individual/L, according to the formula [34]:

(2) $$\;D=\frac{q}{f\times V}$$

where: *D* = zooplankton density (Ind./L), *q* = number of zooplankton found in the subsample (Ind), *f* = fraction taken (subsample volume per sample volume); *V* = volume of filtered water (L)

Plankton wet biomass was measured based on the dimension of cells and cell density for phytoplankton while for zooplankton wet biomass was estimated based on their wet weights and the species density.

### 2.5. Statistical Analysis

In assessing species diversity and water quality conditions, the biodiversity index of Shannon-Weiner (*H*′) [35] was used.

(3) $$H^{\prime }=-\overset{s}{\underset{i=1}{\sum }}\left(\frac{n_{i}}{N}\right)\times \ln\left(\frac{n_{i}}{N}\right)$$

where: *n_i_* = number of individuals or amount (e.g., biomass or density) of each species (the ith species), *N* = total number of individuals (or amount) for the site, ln = the natural log of the number and, s refers to the species type number in a sampling site.

One-way ANOVA was conducted using IBM SPSS Statistics (Version 26.0) to statistically analyze the difference of abundance and biomass of zooplankton and phytoplankton communities between Coal gangue hills sites (S1, S2 and S3), subsidence lake (S4, S5, S6, S7, S8) and Huihe River along with Xiangshun canal (S9, S10, S11, S12).

Correlation coefficients were calculated with the use of Pearson correlation formed in R 4.1.0 and RStudio and RStudio based on the “tidyr” package to describe the degree of relationship between phytoplankton and zooplankton density and water quality parameters, as well as the interaction between phytoplankton and zooplankton taxonomic groups. The graphs of the abundance and biomass values of the plankton were generated with Origin Pro 2018 v 9.5.1 software (OriginLab, Northampton, MA, USA). Heatmaps were created by Prism 9.4.0 (GraphPad).

## 3. Results

### 3.1. Water Characteristics and Physicochemical Parameters

The physicochemical parameters recorded at the sampled sites are summarized in Table S2. Water temperature obtained during the sampling period for all sampling sites ranged between 4.8 and 6 °C, with the highest temperature being observed in the Huihe river sites S10 and S12. The water samples were mainly weakly alkaline in all the sampling points ranging from 7.63 to 8.69. The DO concentrations were in general high in sampling sites ranging between 9.9 mg/L and 12.1 mg/L. The values of Secchi disc depth ranged from 0.8 m in S2 which had a water depth of 1.5 m, to 1.9 m in S4 with a water depth of 4.5 m. TDS, EC, COD_cr_ alongside nutrients concentrations were higher in the coal gangue hill area compared to the Huihe river and subsidence lake. As the highest concentration of TP was marked in S1 with 0.268 mg/L, while the highest values of TN, PO_4_^3−^, NH_4_^+^ were all shown in S3 with 2.71 mg/L, 0.1 mg/L, 0.28 mg/L, respectively.

### 3.2. Phytoplankton Community Characteristics

A total of 50 different phytoplankton species belonging to 7 phyla were identified from the collected samples, where Bacillariophyta, Chlorophyta and Cryptophyta were the most detected taxonomic groups. S2 showed the largest diversity of phytoplankton with a total of 26 species belonging to six phyla being identified.

**Figure 2:**
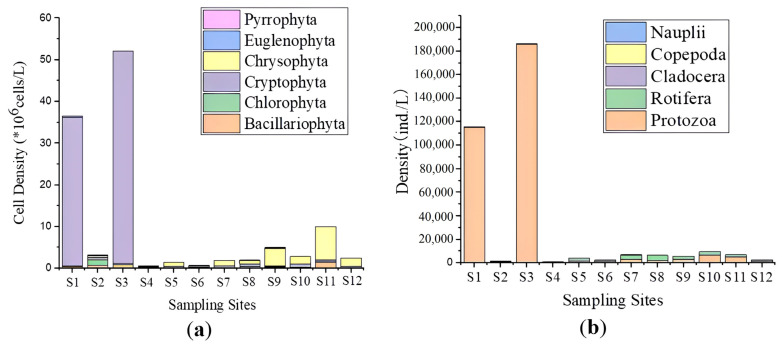
Spatial distribution of plankton abundance in the sampling points. Total density of (**a**) Phytoplankton, (**b**) Zooplankton.

The differences in total cell density of the phytoplankton community between the CGH, SL and HR were significant (*p* < 0.05). The highest total cell density was found at S3 with 52.340 × 10^6^ cell/L followed by S1 with a total density of 36.650 × 10^6^ cell/L where Cryptophyta displayed the highest cell density in both of the sampling points with 50.918 × 10^6^ cell/L and 35.656 × 10^6^ cell/L, respectively, belonging to Cryptophyta phylum. The abundant phytoplankton species in the study areas was *Plagioselmis nannoplanctica*. The lowest total density was found in S4, S6, and S5 with a total density of 0.541 × 10^6^ cell/L, 0.767 × 10^6^ cell/L, 1.485 × 10^6^ cell/L, respectively (Table S3, Figure 2).

**Figure 3:**
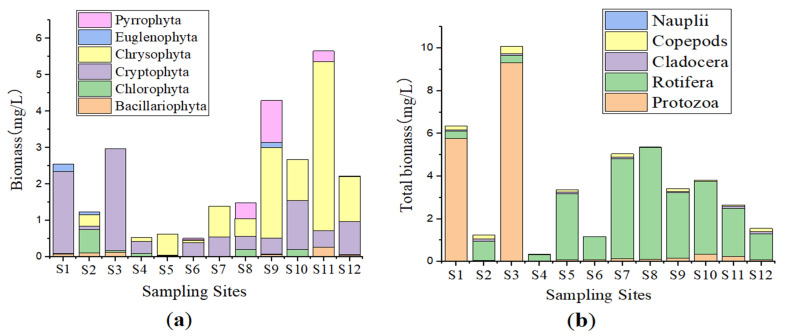
Biomass of plankton community in the sampling points. Total biomass of (**a**) phytoplankton, (**b**) Zooplankton.

Similarly, the Total wet Biomass of the phytoplankton community showed significant variations among the sampling sites (CGH, SL and HR (*p* < 0.05)). It was found to be between 0.5123 mg/L and 5.6532 mg/L; the lowest wet biomass was recorded in S6 while the highest wet biomass value was found in S11 where the Chrysophyta occupied the largest biomass accounting for 4.6546 mg/L (Table S4, Figure 3).

In terms of the biodiversity of the phytoplankton community, the Shannon-Weiner (H’) index showed the highest value at S2 with 2.6, while the lowest values were recorded in S1 and S3 with 0.2 and 0.24 respectively, (Table S5).

### 3.3. Zooplankton Community Characteristics

A total of 41 zooplankton species belonging to 4 phyla (Protozoa, Rotifera, Cladocera, Copepoda) were identified from the collected samples. Protozoa and Rotifera were commonly found and were the most dominant phyla. While species of phyla Cladocera and Copepoda were rarely detected in sampling points, S9 showed the largest diversity of zooplankton with a total of 24 species belonging to the 4 phyla being detected.

Significant differences were observed in the taxa densities of the zooplankton community among the sampling sites (CGH, SL and HR (*p* < 0.05)). The total density ranged from 842.375 ind./L and 186,355.0 ind./L. The total density of the zooplankton community in S3 and S1 was significantly higher than in the other sampling points with 186,355.0 ind./L and 115,529.0 ind./L, respectively, where species of Protozoa phylum (*Strobilidium* sp., *Ciliophora* sp.) occupied the largest portion of the total density in both of the sampling points. The least total density was registered in S4 with a total density of 842.375 ind./L (Table S3, Figure 2).

Non-significant differences were observed in the total wet biomass of the zooplankton community among the sampling points (*p* > 0.05); the total wet biomass ranged between 0.3408 mg/L and 10.0842 mg/L. The highest biomass was observed in S3, while the lowest total biomass was registered in S4. (Table S4, Figure 3).

The biodiversity index of the Shannon-Weiner (H’) of the zooplankton community, varied between the lowest value of 1.18 registered in S1 and the highest value of 2.45 found in S9 (Table S5).

### 3.4. Correlation between Water Quality Parameters and Phytoplankton and Zooplankton

**Figure 4:**
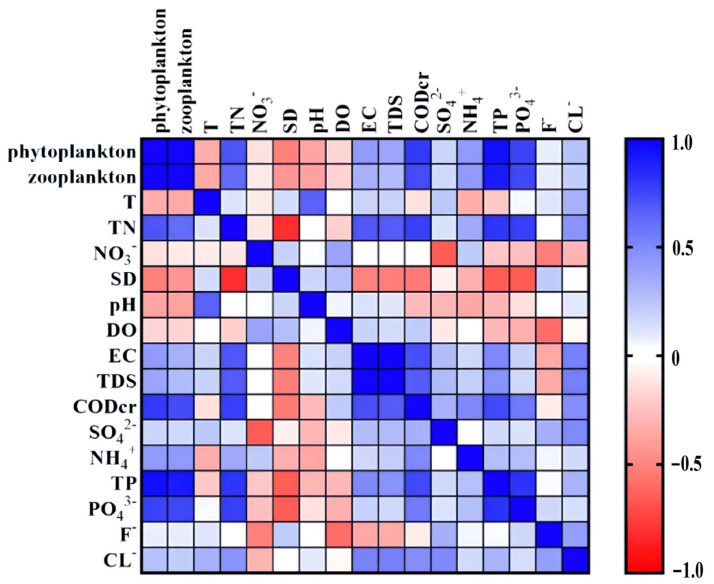
Pearson correlation analysis of the abundance of phytoplankton and zooplankton and water quality variables. The color represents the values of Pearson correlation coefficient.

The Correlation coefficients show how strongly water quality variables are related to the plankton density. The correlation matrix (Table 1, Figure 4) showed a significant correlation of the phytoplankton total density with COD and nutrients in water; TP (correlation: 0.948), PO_4_^3−^ (correlation: 0.763), TN (correlation: 0.701) and with COD_cr_ (correlation: 0.795). Similar to the results of the correlation between phytoplankton communities and water quality factors, the total zooplankton abundance also showed a significant positive correlation with nutrients; TP (correlation: 0.919), PO_4_^3−^ (correlation: 0.745), TN (correlation: 0.613) and with COD_cr_ (correlation: 0.726).

**Figure 5:**
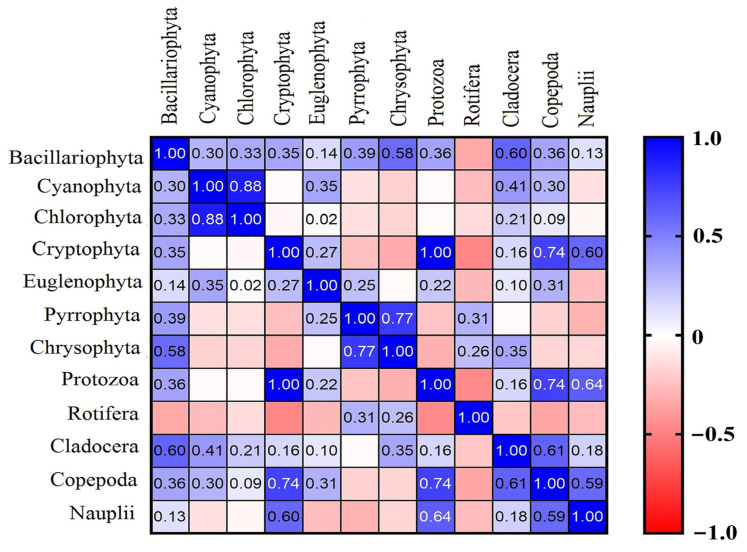
Pearson correlation analysis of the abundance of phytoplankton and zooplankton groups. The color represents the values of Pearson correlation coefficient.

The correlation between the total density of phytoplankton and zooplankton was estimated as a significant positive correlation of 0.987. Most of the zooplankton taxonomic groups showed a non-significant correlation with phytoplankton groups (Table 2, Figure 5), and the correlation matrix revealed the highest correlation was between Protozoa and Cryptophyta with a significant positive correlation of 0.998, followed by Copepoda and nauplii who shared a significant correlation with Cryptophyta 0.736 and 0.596, respectively.

## 4. Discussion

### 4.1. Phytoplankton Characteristics

Many aspects of ecology rely on spatial changes in phytoplankton communities, including the maintenance of species diversity and community stability [36,37]. An assortment of environmental factors and influences shape the community structure, spatial patterns, and abundance of dominant phytoplankton species [26].

Correlation analysis of the plankton communities with physical and chemical water quality factors revealed that nutrients TP, PO_4_^3−^, TN and COD_cr_ were the variables that had the greatest impact on this community. Nutrient availability within waters such as nitrogen or phosphorus are the factors driving the abundance of phytoplankton growth and/or primary productivity. This result is consistent with those of many studies that suggest phosphorus and nitrogen are the limiting nutrients for phytoplankton affecting the different characteristics of phytoplankton community in different water bodies [38,39,40,41], including subsidence lakes [42,43]. The differences in nutrient levels may potentially limit the absorption and utilization of nutrients by phytoplankton and regulate their population abundance and biodiversity [44]. Therefore, excess nutrients lead to excessive growth of phytoplankton, which was shown in the present study as the areas that revealed nutrient pollution, nitrogen, and phosphorus pollution for coal gangue hills area and nitrogen pollution for the Huihe River system; these were the areas that marked the highest total densities of phytoplankton. In addition of the excessive growth of phytoplankton, increased nutrient gradients conduct to decreased phytoplankton diversity [45]. However, even the lack of nutrients may affect the phytoplankton diversity, and it is known that the growth of some phytoplankton species could be restricted by the lack of certain nutrients. Other phytoplankton species with a relatively low demand for these nutrients can rapidly grow and become dominant, resulting in simplification of the species composition and a decrease in biodiversity [44]. This was reflected in the low diversity index in the Huihe River (S11, S10, S12) and Xiangshun canal (S9) as these sites noticed relatively low concentrations of TP.

Organic matter (OM) can play a critical role in advancing the wet biomass and abundance of certain phytoplankton taxa in aquatic environments [46,47], as it can be used by certain phytoplankton as a source of nitrogen and phosphorus [46], which will be reflected in their biodiversity and distribution. Previous studies have demonstrated that the change in phytoplankton structure has a strong correlation with chemical oxygen demand (COD_cr_) and OM [48]. Similarly, in the present study, the COD concentrations stated a significant positive correlation with phytoplankton total density (correlation: 0.795). The excessive concentrations of organic matter in the coal gangue hills (S1, S3), the Huihe River (S11, S10), and Xiangshun canal (S9) worked in favor of the abundance of cryptophytes and chrysophytes, as was the dominant taxa; however, it led to a decrease in the biodiversity in these areas, which was reflected in the uneven distribution of the phytoplankton groups. This suggests that the phytoplankton community in those areas is significantly disturbed.

### 4.2. Zooplankton Characteristics

Zooplankton abundance, distribution, and diversity within aquatic ecosystems are influenced by plenty of factors; biotic such as predation by other aquatic organisms including other zooplankton as well as changes in the phytoplankton communities within the aquatic environment, or abiotic factors which are mainly physicochemical parameters within the aquatic environment [49,50,51].

Similar to [52], Rotifera were dominant as well in the present study, however, Protozoa was the dominant taxa followed by Rotifers as the second dominant taxa. Feeding habits helped to shape the abundance of zooplankton groups in the studied sites. All protozoa require organic materials, whether they be particulate or in solution, as their nutrition is holozoic [53]. Thus, the high concentrations of organic matter favored their growth in all of the investigated sites, especially in coal gangue hills area (S1, S3), which also explains the significant positive correlation between the zooplankton density and COD_cr_. Rotifers play a pivotal role in many freshwater ecosystems. They are ubiquitous, occurring in almost all types of freshwater habitats [54], which explains their presence in all of the sampling points. The high number of rotifers in a freshwater ecosystem is due to their wide variety of feeding habits, high fecundity, and rapid turnover rates [55]. Rotifers feed on protozoa, phytoplankton, detritus, or other organic matter, and they are considered as and are important filter-feeders on algae and bacteria [54,56].

Crustaceans (Cladocera, copepods, and nauplii), as opposed to protozoa and rotifers, were rarely found in all of the sampling points and that is because crustaceans have a longer development cycle, and they are heavier and larger than rotifers [57]. This makes it difficult to maintain crustacean populations in small rivers and even in small reservoirs. Crustaceans occur in rivers with suitable conditions, such as long water retention time, high open water zones, and the presence of macrophytes [58,59,60,61,62,63].

The change in biodiversity is usually related to species dominance [64]. In the present study, the dominance of protozoa and rotifers and the rarity of the crustaceans community reflected the relatively low to moderate biodiversity of the investigated areas.

The decomposition of the organic matter by the dominant phyla Protozoa and Rotifera may potentially explain both the decrease in COD_cr_ and the increase of biodiversity index values of some sampling points in both the waters in the coal gangue hills area (S2) and the Huihe river (S12). However, the subsidence region (subsidence lake and the Coal Gangue Hills) showed less biodiversity than the Huihe River and Xiangshun canal which can provide potential indications of environmental changes or disturbances in those areas, especially in the subsidence water region.

### 4.3. Interactions between Phytoplankton and Zooplankton

Phytoplankton and zooplankton share a predator-prey relationship. Thus, a negative relationship between these two organisms is expected [65]. However, in this study, the total densities of phytoplankton and zooplankton showed a significant positive (very strong) correlation (correlation: 0.987).

Although phytoplankton bloom is closely related to nutrient enrichment, it can be drastically reduced by intensive grazing [27,66,67]. The linkage between phytoplankton and zooplankton is a dynamic process controlled by several factors. Grazing is one of the most important factors controlling the relationship between the two communities as well as zooplankton composition, as the grazing pressure depends on the zooplankton composition since the nature of food selection varies among herbivore taxa [27,68]. Although the grazing causes serious changes in the phytoplankton structure, the bulk of these changes occurs in the dominant species, which are usually grazed more than those that exist in low density or as rarely [27]. In the present study, Protozoa who were the dominant phyla of the zooplankton community, did not show any significant negative correlation with any of the dominant groups of the phytoplankton community (Cryptophyta and Chrysophyta). Similarly, Rotifers who are the second dominant taxa, (although many rotifer species are described as herbivores and microalgae) are the main natural food for rotifers and provide the highest growth and fertility [69]; however, they shared a non-significant correlation with Cryptophyta (correlation: −0.477). Generally, grazing by rotifer-dominated communities is known to be weak, impacting only small cells [70]. This signifies that both Cryptophyta and Chrysophyta seemed to be not preferable by zooplankters during the present study, which lessened the grazing pressure on these phytoplankton phyla and thus favored their growth. This may explain the significant positive correlation between the total density of phytoplankton and zooplankton communities.

Zooplankton appear to have particular impacts on the phytoplankton community, directly through herbivory and indirectly through the recycling of nutrients [71]. Correlation analysis of the zooplankton communities with water quality variables revealed that nutrients TP, PO_4_^3−^ and TN were the variables that were strongly related to zooplankton communities. So, this positive relationship between zooplankton and phytoplankton communities may probably be connected with nutrient release by zooplankton, which stimulates algal growth [72]. Zooplankton communities are important contributors to the nitrogen and phosphorus required by phytoplankton for primary production [73,74], which serves as an additional source of nutrients for phytoplankton [75]. This indicates that phytoplankton growth can be stimulated by zooplankton [64].

## 5. Conclusions

Zooplankton and phytoplankton characteristics and composition may reflect the health of an aquatic ecosystem, both from the point of view of their structural parameters and also of their interrelations with one another. In this context, the present study was undertaken to assess zooplankton and phytoplankton abundance, distribution, and diversity in Linhuan coal mining subsidence area and its surroundings (Huihe River and Xiangshun canal) and the influence of water quality parameters on these communities. The results revealed that both zooplankton and phytoplankton abundance was much higher in the waters of the mining area than in the Huihe River system, while phytoplankton biomass was higher in the Huihe River. Moreover, plankton abundance has been seen to be enhanced by organic and nutrient pollution, giving an additional source of nutrients; however, the ability to use organic nutrients from OM played a role in the distribution in the present study and favored the growth of certain groups (Protozoa and Rotifera for zooplankton and Cryptophyta and Chrysophyta for the phytoplankton community), which is what weakens the diversity of these communities. The interaction between phytoplankton and zooplankton highlighted the selective grazing by zooplankton, as it was among the factors affecting the structure of phytoplankton communities alongside nutrients-release by zooplankton, reflecting a significant positive relationship between these two communities. Both phytoplankton and zooplankton showed less biodiversity in the subsidence water area compared to the Huihe River and Xiangshun canal; however, all these areas revealed disturbed phytoplankton community with a low biodiversity index, while the zooplankton community was more stable in the Huihe River and Xiangshun canal, showing a moderate diversity index. Overall, the findings of the present study provide useful knowledge on the plankton community within the Linhuan subsidence lake as well as the Huihe river area, as it can be used in management strategies to protect the aquatic biodiversity in the subsidence lakes in coal mining areas. Many of the conclusions in this study corroborate the patterns obtained by previous researches. However, some of the different conclusions call for further studies. Therefore, long-term plankton investigations on a spatiotemporal scale are needed to better comprehend the combined effects of physicochemical and biological factors caused by anthropogenic activities, specifically mining activities on both phytoplankton and zooplankton communities in mining areas.

## Figures and Tables

**Table 1:** Correlation coefficient of Pearson correlation of water quality variables, phytoplankton and zooplankton density.

Water Quality Variables	Phytoplankton Density	Zooplankton Density
SD	−0.559	−0.479
COD_cr_	0.795 **	0.726 **
TP	0.948 ***	0.919 ***
PO_4_^3−^	0.763 **	0.745 **
TN	0.701 *	0.613 *
NH_4_^+^	0.424	0.434
SO_4_^2−^	0.179	0.162
NO_3_^−^	−0.135	−0.100
F^−^	0.078	0.083
DO	−0.184	−0.187
T°	−0.336	−0.355
TDS	0.377	0.280
EC	0.423	0.326
pH	−0.375	−0.391
CL^−^	0.264	0.213
Phytoplankton density	1.000	0.987 ***
Zooplankton density	0.987 ***	1.000

2-tailed test of significance is used; Correlation is significant at *p* < 0.05; * *p* < 0.05, ** *p* < 0.01, *** *p* < 0.001.

**Table 2:** Correlation coefficient of Pearson correlation of phytoplankton and zooplankton groups.

	Cyanophyta	Bacillariophyta	Chlorophyta	Cryptophyta	Euglenophyta	Pyrrophyta	Chrysophyta
Protozoa	−0.031	0.364	−0.031	0.998 ***	0.221	−0.238	−0.309
Rotifera	−0.268	−0.338	−0.144	−0.477	−0.282	0.305	0.255
Cladocera	0.405	0.604 *	0.208	0.159	0.095	−0.017	0.351
Copepoda	0.297	0.357	0.086	0.736 **	0.309	−0.178	−0.166
Nauplii	−0.124	0.128	−0.036	0.596 *	−0.264	−0.305	−0.164

The 2-tailed test of significance is used; Correlation is significant at *p* < 0.05; * *p* < 0.05, ** *p* < 0.01, *** *p* < 0.001.

## Data Availability

Not applicable.

## Supplementary Materials

The following supporting information can be downloaded at: https://www.mdpi.com/article/10.3390/ijerph20010484/s1, Table S1. Longitude and latitude coordinates of sampling points; Table S2: Physical and chemical indices of water samples in different sampling points; Table S3: Spatial distribution of plankton abundance in the sampling points; Table S4: Biomass of plankton community in the sampling points; Table S5: Bio-diversity index of plankton community in sampling points.
