# Supplementary material for: Phytoplankton-Zooplankton Community Structure in Coal Mining Subsidence Lake
Source: Int J Environ Res Public Health. 2022 Dec 28;20(1):484. doi: 10.3390/ijerph20010484 (PMC9819133; doi:10.3390/ijerph20010484)
Supplement: Supplementary file 1 [file ijerph-20-00484-s001.zip › ijerph-2048212-supplementary.pdf]

Table S1. Longitude and latitude coordinates of sampling points.

Sampling Point	Latitude	Longitude
S1	N 33°50'56"	E 116°17'43"
S2	N 33°14'42"	E 116°12'21"
S3	N 33°33'03"	E 116°39'44"
S4	N 33°37'07"	E 116°36'38"
S5	N 33°37'12"	E 116°36'59"
S6	N 33°37'15"	E 116°36'59"
S7	N 33°37'13"	E 116°37'14"
S8	N 33°37'23"	E 116°37'10"
S9	N 33°18'6"	E 116°07'14"
S10	N 33°19'32"	E 116°04'55"
S11	N 33°49'23"	E 116°04'88"
S12	N 33°05'40"	E 116°57'79"

Table S2. Physical and chemical indices of water samples in different sampling points.

**Sampling points**	**T ℃**	**TN mg/L**	**NO_3^- mg/L**	**H m**	**SD m**	**pH**	**DO mg/L**	**EC us/cm**	**TDS ppm**	**CODcr mg/L**	**SO_4^{2-} mg/L**	**NH_4^+ mg/L**	**TP mg/L**	**PO_4^{3-} mg/L**	**F^- mg/L**	**CL^- mg/L**
**S1**	4.9	2.42	4.88	2.30	1.00	8.20	10.3	2048	1004	33	136.0	0.00	0.268	0.08	0.356	61.9
**S2**	5.3	2.40	5.66	1.50	0.80	8.33	9.9	2011	1048	20	136.0	0.19	0.126	0.06	0.402	65.2
**S3**	5.0	2.71	6.84	2.70	0.90	8.08	10.7	2046	1015	50	172.0	0.28	0.264	0.10	0.403	62.1
**S4**	5.9	1.22	5.10	4.50	1.90	8.58	10.1	1648	828	12	179.0	0.06	0.048	0.04	0.656	71.8
**S5**	4.8	1.10	7.71	7.00	1.34	7.63	11.1	1582	810	19	183.0	0.11	0.052	0.04	0.324	49.8
**S6**	5.1	1.39	14.70	6.00	1.50	8.57	11.3	1651	827	12	39.8	0.12	0.045	0.04	0.223	42.9
**S7**	5.0	0.87	4.72	3.00	1.64	8.33	11.8	1639	819	12	135.0	0.09	0.055	0.04	0.318	50.5
**S8**	4.8	1.27	4.94	3.50	1.20	8.59	10.5	1652	826	11	137.0	0.08	0.055	0.04	0.380	50.8
**S9**	5.9	1.82	5.94	1.39	1.08	8.62	10.6	2193	1098	20	174.0	0.03	0.097	0.05	0.232	50.2
**S10**	6.0	1.90	8.59	3.64	1.37	8.69	12.0	2146	1073	26	159.0	0.07	0.087	0.05	0.188	69.3
**S11**	5.4	2.29	6.77	5.80	1.10	8.50	12.1	2230	1103	44	158.0	0.13	0.097	0.05	0.345	66.7
**S12**	6.0	1.98	4.67	--	--	8.63	10.5	1482	742	13	136.0	0.04	0.097	0.09	0.436	49.3

Table S3. Spatial distribution of plankton abundance in the sampling points.

		**S1**	**S2**	**S3**	**S4**	**S5**	**S6**	**S7**	**S8**	**S9**	**S10**	**S11**	**S12**
**Zooplankton Density （ind./L）**	**Protozoa***	115200.000	855.000	186000.000	600.000	1560.000	1620.000	2940.000	2040.000	3182.500	6750.000	5005.000	1560.000
	**Rotifera**	300.000	765.000	300.000	240.000	2580.000	900.000	3900.000	4380.000	2565.000	2850.000	1870.000	1020.000
	**Cladocera**	2.400	4.800	3.600	0.475	3.600	0.000	3.000	0.150	1.800	0.600	4.800	4.800
	**Copepoda***	26.400	25.800	49.800	1.900	18.600	1.800	22.200	0.150	22.200	6.600	9.000	22.800
	**Nauplii**	0.000	0.000	1.200	0.000	0.000	0.000	0.000	0.000	0.000	0.600	0.000	0.600
	**Total density***	**115529.000**	**1650.600**	**186355.000**	**842.375**	**4162.000**	**2521.800**	**6865.000**	**6420.300**	**5771.500**	**9608.000**	**6888.800**	**2608.200**
**Phytoplankton cell density (.10^6cells/L)**	**Cyanophyta**	0.201	2.089	0.214	0.000	0.000	0.038	0.063	0.000	0.252	0.000	0.000	0.000
	**Bacillariophyta**	0.478	0.705	0.818	0.025	0.050	0.088	0.013	0.013	0.327	0.050	1.422	0.075
	**Chlorophyta**	0.075	1.258	0.390	0.226	0.013	0.264	0.151	0.453	0.063	0.277	0.289	0.013
	**Cryptophyta***	35.656	0.717	50.918	0.176	0.440	0.252	0.365	0.591	0.189	0.642	0.390	0.377
	**Chrysophyta***	0.000	0.428	0.000	0.113	0.981	0.101	1.397	0.868	4.152	1.887	7.838	2.013
	**Euglenophyta**	0.239	0.113	0.000	0.000	0.000	0.013	0.000	0.000	0.189	0.000	0.000	0.038
	**Pyrrophyta**	0.000	0.000	0.000	0.000	0.000	0.013	0.000	0.038	0.101	0.000	0.075	0.000
	**Total density***	**36.650**	**5.309**	**52.340**	**0.541**	**1.485**	**0.767**	**1.988**	**1.963**	**5.272**	**2.856**	**10.015**	**2.516**

* Indicates significant differences among Coal gangue hills sites (S1, S2 and S3), Subsidence Lake (S4, S5, S6, S7, S8) and Huihe River along with Xiangshun canal (S9, S10, S11, S12) through one-way ANOVA at the 0.05 significance level, $p < 0.05$.

Table S4. Biomass of plankton community in the sampling points

Biomass (mg/L)		S1	S2	S3	S4	S5	S6	S7	S8	S9	S10	S11	S12
Zooplankton	**Protozoa***	5.7600	0.0428	9.3000	0.0300	0.0780	0.0810	0.1470	0.1020	0.1591	0.3375	0.2503	0.0780
	**Rotifera**	0.3600	0.9180	0.3600	0.2880	3.0960	1.0800	4.6800	5.2560	3.0780	3.4200	2.2440	1.2240
	**Cladocera**	0.0480	0.0960	0.0720	0.0095	0.0720	0.0000	0.0600	0.0030	0.0360	0.0120	0.0960	0.0960
	**Copepods***	0.1848	0.1806	0.3486	0.0133	0.1302	0.0126	0.1554	0.0011	0.1554	0.0462	0.0630	0.1596
	**Nauplii**	0.0000	0.0000	0.0036	0.0000	0.0000	0.0000	0.0000	0.0000	0.0000	0.0018	0.0000	0.0018
	**Total wet biomass**	**6.3528**	**1.2374**	**10.0842**	**0.3408**	**3.3762**	**1.1736**	**5.0424**	**5.3621**	**3.4285**	**3.8175**	**2.6533**	**1.5594**
Phytoplankton	**Cyanophyta***	0.0053	0.0219	0.0056	0.0000	0.0000	0.0010	0.0035	0.0000	0.0009	0.0000	0.0000	0.0000
	**Bacillariophyta**	0.0744	0.1124	0.1326	0.0067	0.0067	0.0072	0.0020	0.0007	0.0571	0.0072	0.2691	0.0469
	**Chlorophyta**	0.0160	0.6460	0.0360	0.0910	0.0204	0.0082	0.0006	0.2115	0.0228	0.1931	0.0012	0.0158
	**Cryptophyta**	2.2632	0.0918	2.7969	0.3257	0.0240	0.3824	0.5466	0.3483	0.4317	1.3519	0.4427	0.9161
	**Chrysophyta***	0.0000	0.3074	0.0000	0.1032	0.5699	0.0569	0.8438	0.4940	2.4922	1.1265	4.6546	1.2192
	**Euglenophyta**	0.1910	0.0804	0.0000	0.0000	0.0000	0.0089	0.0000	0.0000	0.1341	0.0000	0.0000	0.0268
	**Pyrrophyta**	0.0000	0.0000	0.0000	0.0000	0.0000	0.0476	0.0000	0.4354	1.1611	0.0000	0.2856	0.0000
	**Total wet biomass***	**2.5498**	**1.2599**	**2.9712**	**0.5265**	**0.6210**	**0.5123**	**1.3965**	**1.4899**	**4.2998**	**2.6787**	**5.6532**	**2.2247**

* Indicates significant differences among Coal gangue hills sites (S1, S2 and S3), Subsidence Lake (S4, S5, S6, S7, S8) and Huihe River along with Xiangshun canal (S9, S10, S11, S12) through one-way ANOVA at the 0.05 significance level, $p < 0.05$.

Table S5. Bio-diversity index of plankton community in sampling points.

Diversity index H'	S1	S2	S3	S4	S5	S6	S7	S8	S9	S10	S11	S12
**Phytoplankton**	0.20	2.60	0.24	1.97	1.17	2.04	1.53	1.52	1.47	1.66	1.40	1.33
**Zooplankton**	1.18	1.90	1.42	1.66	2.00	2.28	2.15	1.72	2.45	2.39	2.36	2.35
